# Supplementary material for: MetaRibo-Seq measures translation in microbiomes
Source: Nat Commun. 2020 Jun 29;11:3268. doi: 10.1038/s41467-020-17081-z (PMC7324362; doi:10.1038/s41467-020-17081-z)
Supplement: Supplementary file 10 — Supplementary Data 7 [file 41467_2020_17081_MOESM10_ESM.zip › File2/Confidence_VeryHigh_Taxonomy/30520_out.krona.html]

Javascript must be enabled to view this page.

members
magnitude
magnitudeUnassigned
count
unassigned
taxon
rank

30520\_out

19

2
superkingdom
12

12
1239
phylum

class
186801
10

186802
order
10


SRS011239\_contig\_number\_22466
1897044
species
1

8
family
541000

946234
genus
1

species
1193534

SRS064276\_contig\_number\_40671
1

genus
1263
1

2293149
species

SRS013965\_contig\_number\_19118
1

1952438
species

SRS019267\_contig\_number\_3794
1


SRS148721\_contig\_number\_49811
1952431
species
1

1898205
species

SRS048060\_contig\_number\_12134SRS049712\_contig\_number\_24187SRS142781\_contig\_number\_2098
3

1924105
genus
1

1
species
1776382

SRS048870\_contig\_number\_13386

1
31979
family

1
1485
genus


SRS147557\_contig\_number\_31354
1637498
species
1

909932
class
1

909929
order
1

1
1843491
family

1
genus
158846


SRS077294\_contig\_number\_contig-100\_1425.95364
437897
species
1


SRS1054691\_contig\_number\_10828
1263019
species
1


SRS015782\_contig\_number\_contig-100\_16269.150667SRS023526\_contig\_number\_contig-100\_13268.137230
2

superkingdom
2157
1

28890
phylum
1

183925
class
1

1
2158
order

2159
family
1

1
2160
genus

SRS893358\_contig\_number\_8447

superkingdom
2759
4

4751
kingdom
4

451864
subkingdom
4

4
4890
phylum

4
147537
subphylum

class
4891
4

order
4892
4

genus
36917
4

4

SRS011239\_contig\_number\_18288SRS011302\_contig\_number\_contig-100\_285.127802SRS020869\_contig\_number\_contig-100\_10057.45783SRS144362\_contig\_number\_contig-100\_2795.197792
species
43984
